# Molecular investigation by whole exome sequencing revealed a high proportion of pathogenic variants among Thai victims of sudden unexpected death syndrome

**DOI:** 10.1371/journal.pone.0180056

**Published:** 2017-07-13

**Authors:** Bhoom Suktitipat, Sakda Sathirareuangchai, Ekkapong Roothumnong, Wanna Thongnoppakhun, Purin Wangkiratikant, Nutchavadee Vorasan, Rungroj Krittayaphong, Manop Pithukpakorn, Warangkna Boonyapisit

**Affiliations:** 1 Department of Biochemistry, Faculty of Medicine Siriraj Hospital, Mahidol University, Bangkok, Thailand; 2 Integrative Computational BioScience Center (ICBS), Mahidol University, Bangkok, Thailand; 3 Department of Forensic Medicine, Faculty of Medicine Siriraj Hospital, Mahidol University, Bangkok, Thailand; 4 Department of Medicine, Faculty of Medicine Siriraj Hospital, Mahidol University, Bangkok, Thailand; 5 Department of Research and Development, Faculty of Medicine Siriraj Hospital, Mahidol University, Bangkok, Thailand; 6 Research Division, Faculty of Medicine Siriraj Hospital, Mahidol University, Bangkok, Thailand; Pennsylvania State University, UNITED STATES

## Abstract

**Introduction:**

Sudden unexpected death syndrome (SUDS) is an important cause of death in young healthy adults with a high incident rate in Southeast Asia; however, there are no molecular autopsy reports about these victims. We performed a combination of both a detailed autopsy and a molecular autopsy by whole exome sequencing (WES) to investigate the cause of SUDS in Thai sudden death victims.

**Materials and methods:**

A detailed forensic autopsy was performed to identify the cause of death, followed by a molecular autopsy, in 42 sudden death victims who died between January 2015 and August 2015. The coding sequences of 98 SUDS-related genes were sequenced using WES. Potentially causative variants were filtered based on the variant functions annotated in the dbNSFP database. Variants with inconclusive clinical significance evidence in ClinVar were resolved with a variant prediction algorithm, metaSVM, and the frequency data of the variants found in public databases, such as the 1000 Genome Project, ESP6500 project, and the Exome Aggregation Consortium (ExAc) project.

**Results:**

Combining both autopsy and molecular autopsy enabled the potential identification of cause of death in 81% of the cases. Among the 25 victims with WES data, 72% (18/25) were found to have potentially causative SUDS mutations. The majority of the victims had at a mutation in the *TTN* gene (8/18 = 44%), and only one victim had an *SCN5A* mutation.

**Conclusions:**

WES can help to identify the genetic causes in victims of SUDS and may help to further guide investigations into their relatives to prevent additional SUDS victims.

## Introduction

Sudden death is one of the common causes of death worldwide. These tragic events can cause medico-legal issues among families and society. It is of great importance to establish the cause of death because of the potential for screening and prophylactic treatment among family members. However, screening autopsy-negative cases, including normal heart and normal toxicology, could be diagnostically challenging. Many of these unexpected deaths can be attributed to lethal arrhythmia disorders, such as hereditary cardiac arrhythmias [1].

Advances in molecular genetics have identified several genes associated with sudden unexpected death. Furthermore, next generation sequencing (NGS), particularly whole exome sequencing (WES), which can rapidly analyze the entire coding sequence of the human genome, has become a cost-effective technique for comprehensive postmortem genomic study. As a result, applying molecular diagnostics as a part of forensic investigation, also known as molecular autopsy, could potentially improve the diagnosis of sudden unexpected death and become crucial to screening family members for prevention [2].

Sudden unexpected death with no structural heart disease is more prevalent in Southeast Asia. The annual incidence was reported at approximately 26–43 per 100,000 in the Thai and Philippine populations compared to approximately 2 per 100,000 in the European population [3–4]. The victims were mostly healthy young males who died at night during sleep. There were no abnormalities found in the autopsy examination that could explain the cause of death. Among the Thai population, the reported cases of sudden unexpected death were mostly from the Northeastern part of Thailand. Several mechanisms have been proposed, including potassium deficiency, renal tubular acidosis, environmental factors, food, and an abnormal autonomic nervous system [5–6]. How those abnormalities contribute to the sudden unexpected death events remains uncertain.

A previous study found that some Thai sudden unexpected death survivors had EKG abnormalities similar to those with Brugada syndrome (BrS) [7]. Further study showed that some sudden unexpected death survivors and families had mutations in *SCN5A* [8], one of the genes associated with BrS. Another study showed that several sudden unexpected death victims from Southern China harbored germline mutations of cardiac sodium channel genes [4]. Based on these data, unexpected death in the Thai population is likely related to hereditary cardiac disorders, especially ion channelopathies. However, genetic studies in Southeast Asian cohorts have been limited to mostly single candidate genes or genotypes [4,9,10]. Here, we performed autopsy and postmortem comprehensive genomic studies by WES for Thai sudden unexpected death cases to determine the prevalence and spectrum of genetic abnormalities that may have been implicated in the pathogenesis of those sudden death events.

## Materials and methods

### Study population

The study was approved by the institutional review board at the Faculty of Medicine Siriraj Hospital. All individuals aged between 18 and 55 years old experienced unexpected death at home and were brought to the hospital for postmortem investigation from January 2015 to August 2015. There was no ante-mortem diagnosis of any medical condition in the victims. All individuals were subjected to autopsy by law. Written informed consents from the responsible relatives were also obtained for further investigation. An autopsy was performed to check for the cause of death according to the guidelines for autopsy practice in sudden death [11–12]. Briefly, a standard autopsy was performed with special attention paid to lesions in the heart and surrounding vascular tissues. Blood, urine, and gall bladder fluid were collected for toxicology screening. Individuals were included into the study if there was deemed to be no cause of death identified from the autopsy, including coronary artery diseases and microscopic myocarditis. Victims found with a positive toxicology report or with poor DNA quality were excluded. The characteristics of all sudden unexpected death syndrome (SUDS) individuals are summarized in Table 1.

**Table 1:** Characteristics of sudden unexpected death syndrome (SUDS) patients.

Variables	Detail	N	%
Sex			
	Male	24	96%
	Female	1	4%
Family history of SUDS		7	28%
Activity at time of death			
	Sleeping	22	88%
	At rest or light activity	3	12%
Region of origin in Thailand			
	Central	9	36%
	Northeastern	12	48%
	Western	2	8%
	Southern	0	0%
	Northern	2	8%
Time of death			
	06:01–12:00	4	16%
	12:01–18:00	2	8%
	18:01–00:00	6	24%
	00:01–06:00	13	52%
History of syncope		1	4%
Alcohol consumption		7	28%
Smoking		8	32%
Amphetamine Use		1	4%
BMI (Mean ± SD)		22.93 ± 3.21	
Age (Mean ± SD)		31.04 ± 13.12	
Heart weight (Mean ± SD)		338.75 ± 79.96	
Left lung weight (Mean ± SD)		560.8 ± 155.19	
Right lung weight (Mean ± SD)		694.8 ± 200.21	
Brain weight (Mean ± SD)		1357.2 ± 96.76	

### DNA extraction and whole exome sequencing

Blood samples were obtained from the deceased individuals during autopsy. DNA was extracted using an automated DNA extraction platform (chemagic MSM I instrument, PerkinElmer chemagen Technologie GmbH, Baesweiler, Germany). DNA samples were diluted with 10 mM Tris-HCl and 0.1 mM EDTA buffer to a concentration of 100 ng/μL before sending them out for exome sequencing. The exome enrichment library was constructed using the Agilent SureSelect^XT^ version 5 kit (Agilent Technologies, Santa Clara, CA, USA). The library preparation protocol was supplied by the manufacturer. The TruSeq 3000/4000 SBS Kit v3 was used as a reagent for paired-end sequencing with a read length of 101 bp on the Illumina HiSeq 4000. The details of the sequence data processing and variant calling are available in the online supplementary methods.

### Candidate genes for SUDS

We curated a list of 98 possible candidate genes from the literature [13–15] (S1 Table). These are the genes that have been reported to cause BrS, SUDS from ventricular arrhythmia, cardiac channelopathies or cardiomyopathy. Variants called by *GATK* [16] that fall within these genes were extracted for further analysis.

### Candidate variant filtration

The putative impact of the variant types was classified according to *SNPEff v*.*4*.*1L* Software as high, moderate, low or modifier according to the variant’s functional annotation in sequence ontology terms [17]. For example, genetic changes that are classified in the “high impact” group include abnormal chromosome number, variants causing an exon loss, a shift in the reading frame of the encoded protein, rare amino acid variants, transcript ablation, or alteration of the mRNA splice sites or the start or stop codon of the protein.

To obtain a list of potentially disease-causing variants, we first exclude known variants reported to be likely benign, benign, or with other functional relationships (e.g., confers sensitivity, risk factors, association, protective, affects) according to the ClinVar database's clinical significance. Variants overlapping with multiple transcripts were annotated based on the most deleterious functional changes. *MetaSVM* was then used to filter out any variants predicted to be tolerable [18]. Lastly, the frequency of variants reported in publicly available population databases was used to exclude common variants with more than 10% allele frequency in any reference population available.

### Thai WES control

The frequencies of variants identified as potentially causative variants were checked against the whole exome sequencing data in 162 non-SUDS Thai patients [19, 20].

## Results

### Prevalence of SUDS

**Fig 1:**
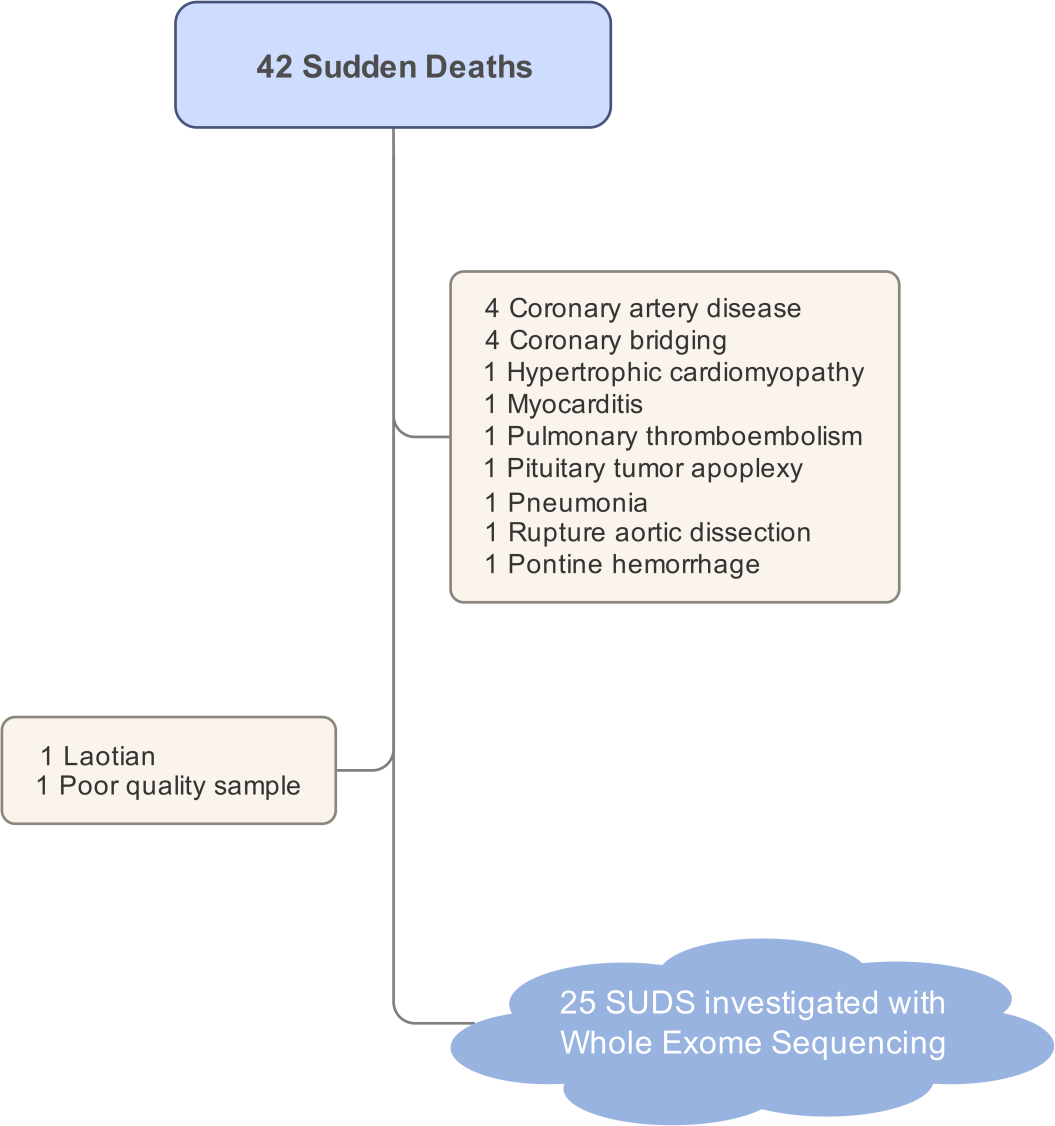
A summary of all sudden death victims. Autopsy identified the cause-of-death in 14 victims. The remaining 25 sudden unexplained death (SUDS) victims were investigated with whole exome sequencing.

Forty-two individuals with sudden deaths were included in the study. Twenty-seven individuals (64%) with no identifiable cause of death from autopsy were classified as SUDS. One Laotian and one individual with poor DNA quality were excluded from the study (Fig 1).

The majority of individuals with SUDS were male (96%), with an average age of 31 years (range from 21 to 51 years). Most individuals died during sleep (88%). They were mostly from the northeastern (48%) and central (36%) parts of Thailand. A family history of SUDS in a first-degree relative present in 28% of cases. The characteristics of all patients are summarized in Table 2.

**Table 2:** Characteristics of each patient.

StudyID	Age	Sex	Time of death*	Hometown †	Activity at time of death	Underlying disease	Family history	BMI	Gene identified ^‡^
SCD-002	40	M	24.00–6.00	Central	Sleeping	No	Yes	25.00	*TTN*, *ILK*, *TMP1*
SCD-004	29	M	6.00–12.00	Central	At rest	No	No	20.38	*—*
SCD-009	47	M	18.00–24.00	NE	Sleeping	No	No	17.15	*—*
SCD-010	48	M	6.00–12.00	NE	Sleeping	No	Yes	18.07	*MYH7*, *TTN*
SCD-011	33	M	24.00–6.00	NE	Sleeping	No	No	22.50	*TPM1*
SCD-013	23	M	18.00–24.00	Central	At rest	No	No	18.69	*CACNB2*, *MYBPC3*
SCD-014	38	M	24.00–6.00	NE	Sleeping	No	No	20.96	*—*
SCD-015	25	M	18.00–24.00	NE	Sleeping	No	No	20.90	*DSP*, *KCNQ1*
SCD-017	30	M	24.00–6.00	NE	Sleeping	No	No	22.57	*SCN5A*, *KCNH2*, *MYLK2*
SCD-018	30	M	24.00–6.00	NE	Sleeping	No	Yes	29.75	*TNNT2*, *TTN*
SCD-019	37	M	24.00–6.00	Central	Sleeping	No	No	25.51	*TTN*
SCD-020	42	M	7.00	NE	Sleeping	No	Yes	23.23	*NEXN*
SCD-025	37	M	12.00–18.00	Central	Sleeping	No	No	25.10	*RYR2*, ***KCNQ1***
SCD-026	33	M	3.30	Central	Sleeping	No	Yes	24.38	*BAG3*
SCD-029	47	M	24.00–6.00	Central	Sleeping	No	Yes	18.83	*TTN*
SCD-032	34	M	24.00–6.00	W	sleeping	No	No	28.34	*KCNQ1*
SCD-034	34	M	18.00–24.00	NE	Sleeping	No	No	22.50	*—*
SCD-035	51	M	22.00	Central	Sleeping	No	No	22.66	*TTN*
SCD-036	35	M	5.30	Central	Sleeping	No	No	26.13	*—*
SCD-037	21	M	10.14	NE	Sleeping	No	NA	20.96	*—*
SCD-038	25	M	15.00	NE	Sleeping	No	Yes	25.61	*CACNB2*, *KCNH2*
SCD-039	25	M	24.00–06.00	W	Sleeping	No	NA	26.22	*TTN*
SCD-040	39	M	22.25	NE	At rest	No	NA	25.59	*RBM20*
SCD-041	34	M	24.00–06.00	NE	Sleeping	No	No	20.52	*—*
SCD-042	29	F	24.00–06.00	NE	Sleeping	Syncope	No	21.64	*TTN*, ***KCNH2***, *CACNA1C*

* Exact time of death or the interval

† Region in Thailand

^**‡**^ Underlined gene = double heterozygote

bolded gene = homozygote

### Whole exome variant filtering

**Fig 2:**
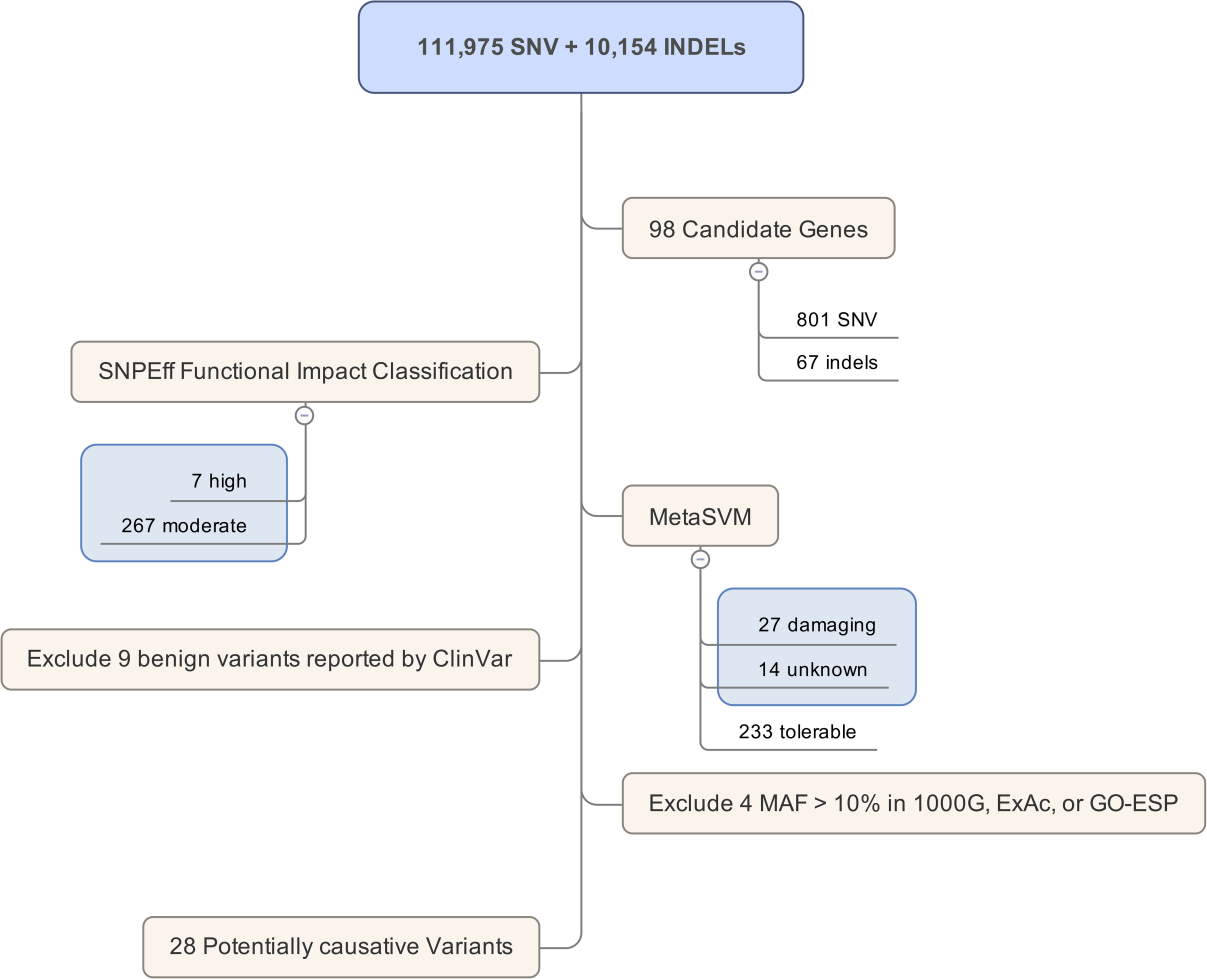
A summary result of whole exome variant filtering. (SNV = single nucleotide variants, MAF = minor allele frequency).

From WES, we identified 111,975 variants and 10,154 small indels among all 25 individuals. After filtering by the 98 candidate gene regions, 868 variants (801 SNPs and 67 indels) were found within the genes previously reported to cause BrS, ventricular arrhythmia, or cardiomyopathy. Using the functional classification of the variants, 274 had a high or moderate impact on the gene function. Of these, 233 were predicted by metaSVM to be tolerable, 9 were reported by ClinVar to be benign, and 4 were common variants found in public frequency databases (Fig 2). In summary, 28 potentially causative variants were identified in 18 individuals (72%) (Tables 3 and 4).

**Table 3:** Potentially causative variants in SUDS.

	Gene	CHR	POS	rsID	REF	ALT	MetaSVM	Effect	1000G	ExAC	ESP	THAI	StudyID
1	NEXN	1	78383329	rs200345240	G	A	D	p.Glu36Lys	0.0002	0.00002	-	-	20
2	TNNT2	1	201331239	-	G	A	-	p.Ala192Val	-	-	-	-	18
3	RYR2	1	237754226	rs373261115	C	T	D	p.Ala1365Val	0.0016	0.0003	0.00008	0.0093	25
4	TTN	2	179418854	rs187460377	C	T	D	p.Gly29662Ser	0.0002	0.0002	0.00008	-	29
5	TTN	2	179426553	-	C	A	D	p.Trp28102Cys	-	-	-	-	19
6	TTN	2	179438185	rs55992239	G	A	D	p.Pro22584Leu	0.0002	0.00007	0.0002	0.0038	18
7	TTN	2	179484555	rs371299188	C	A	D	p.Val13856Phe	0.0006	0.0001	-	-	39, 42
8	TTN	2	179629493	rs55634230	A	C	D	p.Val3250Gly	0.0004	0.00006	-	-	10, 35
9	TTN	2	179640850	rs374203813	G	A	D	p.Ala1914Val	0.0002	0.00002	-	-	2
10	SCN5A	3	38662409	-	C	T	D	p.Arg179Gln	-	0.00003	-	-	17
11	DSP	6	7580187	-	G	C	D	p.Arg1255Thr	-	0.00002	-	-	15
12	KCNH2	7	150644474	rs373394254	G	A	D	p.Arg1032Trp	0.0004	-	-	-	17
13	KCNH2	7	150646128	-	T	G	D	p.Asp463Ala	-	-	-	-	42
14	KCNH2	7	150649611	rs562875924	C	T	D	p.Gly487Ser	0.0004	0.00006	-	-	38
15	CACNB2	10	18828275	-	AG	A	-	frameshift	-	-	-	-	38
16	CACNB2	10	18828310	rs377657305	G	A	D	p.Arg492His	0.0004	0.00008	0.0002	-	13
17	RBM20	10	112590879	rs371181124	C	T	D	p.Thr1171Met	-	-	0.0002	-	40
18	BAG3	10	121429499	-	G	A	D	p.Arg106Gln	-	-	-	-	26
19	KCNQ1	11	2591954	-	C	T	D	p.Arg192Cys	-	0.00003	-	-	15
20	KCNQ1	11	2869129	rs1800172	G	A	D	p.Gly516Ser	0.0166	0.0167	0.0069	-	32
21	ILK	11	6621766	rs142644288	G	A	-	p.Arg401*	-	0.00003	0.00008	-	2
22	MYBPC3	11	47364415	-	A	T	D	p.Cys475Ser	-	-	-	-	13
23	CACNA1C	12	2675634	rs371702432	G	A	D	p. Ala519Thr	-	0.00006	0.00008	-	42
24	MYH7	14	23891399	rs192722540	G	A	D	p.Arg1079Trp	0.0004	0.00005	-	-	10
25	TPM1	15	63353987	-	G	GTACTCTCAGAAGGAAGACAAATATGAAGAGGAGATCAAGGTTCTCTCTGACAAGCTGAAGGAGGCTGAAACTC	-	splice variant	-	-	-	0.018	11
26	TPM1	15	63354780	-	G	GAAGTACTCTCAGAAGGAAGACAAATATGAAGAGGAGATCAAGGTTCTCTCTGACAAGCTGAAGGAGGCTGAA	-	p.Glu236_Thr237ins	-	-	-	-	2
27	SCN1B	19	35524607	rs72558029	G	A	D	p.Val138Ile	0.0210	0.0117	0.0002	-	18, 35
28	MYLK2	20	30419883	-	C	T	-	p.Arg552*	-	-	-	-	17

CHR/POS = chromosome and position (genome build GRCh37), rsID = dbSNP ID, REF/ALT = Reference/alternative allele, MetaSVM (D) = deleterious variant predicted by MetaSVM; AF = allele frequency; 1000Gp = AF in 1000 Genome Project phase 3, ExAC = AF in Exome Aggregation Consortium, GO-ESP = AF in NHLBI Exome Sequencing Project, THAI = AF in Thai control group, (AF data in1000G, ExAC, ESP from dbSNP: https://www.ncbi.nlm.nih.gov/projects/SNP/)

**Table 4:** Potential pathogenic variants in each patient.

		Patient ID (SCD-ID)																								
Gene	Effect	02	04	09	10	11	13	14	15	17	18	19	20	25	26	29	32	34	35	36	37	38	39	40	41	42
*NEXN*	p.Glu36Lys												***1***													
*TNNT2*	p.Ala192Val										***1***															
*RYR2*	p.Ala1365Val													***1***												
*TTN*	p.Gly29662Ser															***1***										
*TTN*	p.Trp28102Cys											***1***														
*TTN*	p.Pro22584Leu										***1***															
*TTN*	p.Val13856Phe																						***1***			***1***
*TTN*	p.Val3250Gly				***1***														***1***							
*TTN*	p.Ala1914Val	***1***																								
*SCN5A*	p.Arg179Gln									***1***																
*DSP*	p.Arg1255Thr								***1***																	
*KCNH2*	p.Arg1032Trp									***1***																
*KCNH2*	p.Asp463Ala																									***2***
*KCNH2*	p.Gly487Ser																					***1***				
*CACNB2*	frameshift																					***1***				
*CACNB2*	p.Arg492His						***1***																			
*RBM20*	p.Thr1171Met																							***1***		
*BAG3*	p.Arg106Gln														***1***											
*KCNQ1*	p.Arg192Cys								***1***																	
*KCNQ1*	p.Gly516Ser													***2***			***1***									
*ILK*	p.Arg401*	***1***																								
*MYBPC3*	p.Cys475Ser						***1***																			
*CACNA1C*	p. Ala519Thr																									***1***
*MYH7*	p.Arg1079Trp				***1***																					
*TPM1*	splice variant					***1***																				
*TPM1*	p.Glu236_Thr237ins	***1***																								
*SCN1B*	p.Val138Ile										***1***								***1***							
*MYLK2*	p.Arg552*									***1***																

The numbers showed in each column of patient ID represent the number of genetic variant at each locus: 1 = heterozygous, and 2 = homozygous

## Discussion

### Overall findings

This is the first study to perform a molecular autopsy by WES in SUDS victims in Southeast Asia, which is an endemic area of SUDS. In this study, we investigated 42 Thai SUDS victims, 25 of which were without a definite cause of death from autopsy and underwent comprehensive genomic investigation to identify variants in genes associated with hereditary cardiac arrhythmias. The victims’ characteristics were similar to those with SUDS in Southeast Asia [21]. A positive family history of SUDS supported that these victims could suffer from the same condition, though we were not able to obtain a definite cause of death from those families.

### Analytical strategy for variant filtering

Following the commonly used strategy, which focuses on the known functional effects of variants in the candidate genes [22–23], we chose to filter down the list of potentially causative variants in SUDS victims based mainly on the known causative genes reported in the literature [13–15]. Twenty-eight potentially causative variants in genes associated with sudden cardiac death (SCD) were identified in 76% of SUDS victims (Fig 2 and Table 3). The rate of molecular diagnosis is relatively higher than the average 25% reported in unknown disease groups undergoing WES [24]. Hence, having a provisional diagnosis of the disease before performing WES to confirm the diagnosis can help to improve the diagnostic yield of the disease. The rate of identification of the genetic cause was quite similar to that of the cohort of natural death and SUDS undergoing post-mortem genetic investigation of 55 genes related to SCD, which reported a molecular diagnostic rate of 40–50% [25].

### Application of metaSVM

The prediction from metaSVM can help to resolve the inconsistent evidence provided from other resources, such as ClinVar, as summarized in S2 Table (meta-ClinVar = x|5). All three missense mutations have been reported in the ClinVar database with inconclusive evidence as both pathogenic variants and variants of unknown significance; however, none of these variants were predicted by metaSVM to be deleterious. One of these variants, rs1805124 (p.His558Arg) in *SCN5A*, has been reported as a pathogenic variant for SUDS in the ClinVar database. However, rs1805124 appears to be a common variation found in all populations with a frequency over 20%. metaSVM predicted the change from rs1805124 to be tolerable. Another variant in *SCN5A*, rs41261344 (p.Arg1192Gln), was also predicted to be tolerable by the metaSVM algorithm. The pathogenicity report of rs41261344 in ClinVar was inconsistent. Therefore, we excluded these two variants in *SCN5A* from the final list of potentially causative variants in our SUDS samples (Table 3). In contrast, another variant in *SCN5A*, NM_001099404.1 c.536G>A, p.Arg179Gln, was a novel variant that has not been reported before. Furthermore, this variant was predicted to be damaging. Hence, we kept this variant in the list of potentially causative variants.

### *TTN* and *SCN5A* in SUDS

In total, we have identified 32% (8/25) of the SUDS victims as having mutations in *SCN5A*: 1 novel variant, 5 with rs1805124 and 2 with rs41261344. However, as we excluded the two non-pathogenic variants in *SCN5A*, only 1 out of 25 (4%) SUDS victims could be explained by the mutation in *SCN5A*. The frequency of *SCN5A* mutations in our sample was quite different from the number reported previously among a BrS cohort, which estimated the frequency of *SCN5A* mutations to be from 11% to 28% [26]. One possible explanation could be that *SUDS* is a complex syndrome, with BrS type I partially contributing to SUDS. Considering the other genes related to BrS, there are 5 out of 25 (25%) that might be affected by BrS of various types.

Interestingly, 8 out of 25 (28%) of the SUDS victims in our study had at least one potentially pathogenic variant in *TTN* that might either cause or modify the risk of SUDS. The clinical significance of these variants remained unclear. However, our results suggest that screening for *TTN* mutations could improve the molecular diagnosis rate among SUDS victims. A previously published report of rare variants in *TTN* in over 1,126 SCD victims, composed of patients with channelopathies, cardiomyopathies, and SUDS, identified 554 potentially causative variants [27]. However, no *TTN* variants reported in this study overlapped with the list of variants reported previously, which might suggest different allelic heterogeneity in *TTN* contributing to SCD in the Spanish population versus in the Thai population.

### Clinical implications

Several reports of SUDS among Asian populations have suggested that the cause of death may be related to a sleep-related mechanism or coronary spasm [28]. The identification of genetic variants among sudden death victims confirmed that hereditary cardiac arrhythmias significantly contribute to the pathogenesis of SUDS in Thailand. Overall, the diagnostic yield from our study was remarkably higher than that of other sudden death reports, which typically range from 10% to 40% [29–30]. Three factors may explain the diagnostic yield difference. First, our cohort was composed of all young Thai individuals with clinical characteristics similar to the SUDS that is prevalent in Southeast Asia. This condition has a genetic influence with multiple causative mutations that have been identified previously. Second, the high family history of SUDS suggested that some tragedies were from inherited conditions. Third, the comprehensive genetic study that covered more associated genes provided a higher chance of detecting causative variants than earlier reports that focused on a limited set of genes.

WES greatly expands genetic investigations from a few genes to the entire coding sequences of the human genome and improves diagnostic yield. However, the large amount of sequence data also creates a significant challenge for data analysis and interpretation, especially when variants are found in genes not known to be associated with SUDS. The uncertain findings potentially make the counseling process difficult for victims’ relatives. Therefore, it is more appropriate to target only variants found in a panel of genes associated with SUDS.

A molecular autopsy can help to determine the cause of death and leads to precise screening methods and clinical management in at-risk relatives, such as lifestyle modification or cardioverter defibrillator implantation that can prevent the occurrence of SUDS in family members. WES will lead to the analysis of known cardiac-related causative genes and to potential new gene discovery. Determining the pathogenicity of the variants identified in the molecular autopsy is complicated by the absence of a phenotype in the victims. We rely on the characteristics of the variant, frequency in populations, previous reports, any available functional data, and overall predictions from *in silico* tools. Finding a pertinent clinical cardiac phenotype in family relatives of the victims may help in the determination of the pathogenicity of the genetic variants identified in SUDS.

### Limitations

Among the victims with sudden death, the initial detailed autopsies were able to identify the cause of death, such as coronary artery disease, abnormal coronary anatomy, myocarditis, aortic dissection, or pulmonary thromboembolism, in 36% of the cases. If we included the victims who died from those identifiable causes, the molecular autopsy yield might be lower than the 72% reported here. Furthermore, we would be introducing bias and genetic heterogeneity to the cause of SUDS in our study had we included patients who died from other causes. Hence, a careful and detailed autopsy is a very crucial step to minimize genetic heterogeneity in the samples and can help to improve the chance to identify potentially causative variants.

With a sample limited to the SUDS victims in our study, we were unable to estimate the effects of the reported variants in the general population. With a large sample of the general population in Thailand, WES would enable us to investigate the presence of these potentially causative variants in the general population. However, given that these variants with predicted damaging effects are mostly novel or rare in the general population including Thais, the variants reported here should be promising causative variants of SUDS.

## Conclusion

A significant proportion of Thai victims with SUDS is caused by various inherited genetic abnormalities, especially in young individuals with no abnormal structural heart diseases by autopsy. Genetic investigation by NGS provides an affordable and powerful diagnostic tool in SUDS and could prevent further loss through family screening and lifesaving management among victims’ family members.

## Supporting information

S1 Table List of genes related to SUDS. (XLSX)

S2 Table List of variants with high and moderate impact on function. (XLSX)

S1 File Supplementary method. (DOCX)
