# Supplementary material for: Molecular investigation by whole exome sequencing revealed a high proportion of pathogenic variants among Thai victims of sudden unexpected death syndrome
Source: PLoS One. 2017 Jul 13;12(7):e0180056. doi: 10.1371/journal.pone.0180056 (PMC5509116; doi:10.1371/journal.pone.0180056)
Supplement: S1 File — (DOCX) [file pone.0180056.s003.docx]

## Exome sequence data processing and variant filtering

Short reads obtained from sequencing as fastq files were mapped to reference genome GRCh37 using *bwa v.0.7.5a* software ^1^. Aligned reads were marked for duplications, and local realignment of small indels was performed utilizing Genome Analysis Tool Kit (GATK) *v.3.3.0* ^2,3^ with *Mills_and_1000G_gold_standard.indels.b37.sites.vcf* and *1000G_phase1.indels.b37.vcf* obtained from the *GATK resource bundle* website (<https://www.broadinstitute.org/gatk/download/>) as the known indel locations ^4^. The *HaplotypeCaller* function of *GATK* was used to simultaneously call both single nucleotide polymorphisms (SNPs) and indels. An intermediate genomic *gVCF* file was created for each sample, which was subsequently used for joint genotyping from all 25 patients to obtain the correct genotype likelihood. Quality control of the variants was performed to keep the variants with Quality by Depth (QD) > -2, Mapping Quality (MQ) > 30, Fisher's test for Strand bias (FS) ≤ 40, Mapping Quality Rank Sum Test (MQRankSum) > -12.5, Read Position Rank Sum Test (ReadPosRankSum) > -8, and Depth per Sample (DP) > 8. Annotation was performed using *SnpEff v.4.1L* software ^5^. The variants were annotated through *SnpEff* using the frequency data from the 1000 genome project phase 1^6^, the Exome Aggregation Consortium (ExAC) project ^7^, and the NHLBI Exome Sequencing project (ESP6500) ^8,9^. The clinical significance of each variant was based on the ClinVar database (freeze 20140902). The main functional prediction data were annotated through *SnpEff* using [dbNSFP v2.9](https://sites.google.com/site/jpopgen/dbNSFP) as the reference data source ^10^. Additional information about the variants can be found in the functional annotation database, i.e., the dbNSFP database ^10^, which has integrated several sources of information, including ClinVar ^11^. dbNSFP has curated over 89 million non-synonymous single nucleotide variants and splice site variants together with functional prediction from other software, such as SIFT, Polyphen2, MutationTaster, and metaSVM. We used the metaSVM ensemble scores, which aggregate prediction information from other functional prediction software and combine the information to create an ensemble score ^12^, instead of counting the number of consensus predictions and using the majority of the results to justify the deleteriousness of the variants.

From WES, we identified 111,975 variants and 10,154 small indels among all 25 individuals. After filtering by the 98 candidate gene regions, 868 variants (801 SNPs and 67 indels) were found within the genes previously reported to cause BS, ventricular arrhythmia, or cardiomyopathy. From this list of variants, 274 variants were classified by *SNPEff* to have a high or moderate impact on the protein function (Supplementary Table 1). The 7 loci with *high* impacts on the protein function caused frameshift mutation indels (4), stop gain codons (2), and splice donor variants (1). The additional 267 loci were classified as having moderate impact on the gene functions. We chose to discard 774 variants that had low impact or were classified as modifiers.

We further filtered out non-synonymous mutations that may not affect the function of the gene. Several types of functional prediction information were annotated from the dbNSFP database. However, we predicted the role of missense variants with *MetaSVM,* which integrated the functional predictions from other popular prediction algorithms ^12^. Variants predicted to be tolerable by *metaSVM* were filtered out. Examples of the excluded variants are rs1805124 in *SCN5A* and rs12720449 in *KCNQ1*. These two variants were found in 5 and 7 patients, respectively. The frequencies of rs1805124 were relatively common in the 1000 Genomes Project, ExAC, and ESP6500 databases (all exceeding 20%). rs12720449 was relatively uncommon, with an allele frequency of less than 5% in all of the public databases mentioned. Both rs1805124 and rs12720449 were predicted to be tolerable; therefore, both variants were excluded from the final results. Using *metaSVM* to exclude tolerable variants, 41 variants remained either predicted to be damaging or with no known prediction score from *metaSVM*.

The ClinVar database (Update 12/05/2017) was used to further filter out 9 variants classified as benign or likely benign (https://www.ncbi.nlm.nih.gov/clinvar/docs/clinsig/). Out of 274 variants with high or moderate impact functional classification, were reported as benign or likely benign (148), uncertain significance (23), and unknown or other significant (68), or presented with conflicting evidence (37)in their ClinVar classification (Supplementary Figure 1). Two variants, rs200371894 in *MAP2K2* and rs3729712 in *TNNI3*, were reported to be likely benign in ClinVar, and were excluded from the final report. However, these two variants were predicted to be deleterious by metaSVM algorithm. We further checked to see the frequencies of these variants in the general population as the next step.

Finally, the frequencies of the remaining variants were updated from the 1000 Genomes Project Phase 3 data and from the GO-ESP database, directly queried from NCBI’s dbSNP database. We identified 4 common variants reported with an allele frequency greater than 10%, which were excluded. Although these variants were predicted to be deleterious with an *in silico* prediction algorithm, we chose to exclude them from the list of potentially causal variants in SUDS as the prevalence of SUDS in the general population is rare. Supplementary Figure 1 summarizes the main results from our variant filtration algorithm.

### Reference

1. Li H, Durbin R: Fast and accurate short read alignment with Burrows–Wheeler transform. Bioinformatics [Internet] 2009 [cited 2017 Jan 12]; 25:1754–1760. Available from: http://www.ncbi.nlm.nih.gov/pmc/articles/PMC2705234/

2. McKenna A, Hanna M, Banks E, Sivachenko A, Cibulskis K, Kernytsky A, Garimella K, Altshuler D, Gabriel S, Daly M, DePristo MA: The Genome Analysis Toolkit: A MapReduce framework for analyzing next-generation DNA sequencing data. Genome Res [Internet] 2010 [cited 2017 Jan 12]; 20:1297–1303. Available from: http://genome.cshlp.org/content/20/9/1297

3. DePristo MA, Banks E, Poplin R, et al.: A framework for variation discovery and genotyping using next-generation DNA sequencing data. Nat Genet [Internet] 2011 [cited 2017 Jan 12]; 43:491–498. Available from: http://www.nature.com/ng/journal/v43/n5/full/ng.806.html

4. Van der Auwera GA, Carneiro MO, Hartl C, et al.: From FastQ data to high confidence variant calls: the Genome Analysis Toolkit best practices pipeline. Curr Protoc Bioinforma Ed Board Andreas Baxevanis Al [Internet] 2013 [cited 2017 Jan 12]; 11:11.10.1-11.10.33. Available from: http://www.ncbi.nlm.nih.gov/pmc/articles/PMC4243306/

5. Cingolani P, Platts A, Wang LL, Coon M, Nguyen T, Wang L, Land SJ, Lu X, Ruden DM: A program for annotating and predicting the effects of single nucleotide polymorphisms, SnpEff. Fly (Austin) [Internet] 2012 [cited 2017 Jan 5]; 6:80–92. Available from: http://www.ncbi.nlm.nih.gov/pmc/articles/PMC3679285/

6. The 1000 Genomes Project Consortium: A global reference for human genetic variation. Nature [Internet] 2015 [cited 2016 Apr 10]; 526:68–74. Available from: http://www.nature.com/nature/journal/v526/n7571/full/nature15393.html

7. Lek M, Karczewski KJ, Minikel EV, et al.: Analysis of protein-coding genetic variation in 60,706 humans. Nature [Internet] 2016 [cited 2017 Jan 12]; 536:285–291. Available from: http://www.nature.com/nature/journal/v536/n7616/full/nature19057.html

8. Auer PL, Reiner AP, Wang G, Kang HM, Abecasis GR, Altshuler D, Bamshad MJ, Nickerson DA, Tracy RP, Rich SS, Leal SM: Guidelines for Large-Scale Sequence-Based Complex Trait Association Studies: Lessons Learned from the NHLBI Exome Sequencing Project. Am J Hum Genet [Internet] 2016 [cited 2017 Jan 12]; 99:791–801. Available from: http://www.sciencedirect.com/science/article/pii/S0002929716303378

9. Tennessen JA, Bigham AW, O’Connor TD, et al.: Evolution and Functional Impact of Rare Coding Variation from Deep Sequencing of Human Exomes. Science [Internet] 2012 [cited 2017 Jan 12]; 337:64–69. Available from: http://science.sciencemag.org/content/337/6090/64

10. Liu X, Jian X, Boerwinkle E: dbNSFP v2.0: A Database of Human Non-synonymous SNVs and Their Functional Predictions and Annotations. Hum Mutat [Internet] 2013 [cited 2015 Jul 18]; 34:E2393–E2402. Available from: http://onlinelibrary.wiley.com/doi/10.1002/humu.22376/abstract

11. Landrum MJ, Lee JM, Benson M, et al.: ClinVar: public archive of interpretations of clinically relevant variants. Nucleic Acids Res [Internet] 2016 [cited 2017 Jan 6]; 44:D862–D868. Available from: http://nar.oxfordjournals.org/content/44/D1/D862

12. Dong C, Wei P, Jian X, Gibbs R, Boerwinkle E, Wang K, Liu X: Comparison and integration of deleteriousness prediction methods for nonsynonymous SNVs in whole exome sequencing studies. Hum Mol Genet [Internet] 2015 [cited 2016 Dec 6]; 24:2125–2137. Available from: http://hmg.oxfordjournals.org/content/24/8/2125
